# Different medical expenses incurred by appendiceal cystadenoma in China: Report of three cases

**DOI:** 10.3892/ol.2013.1174

**Published:** 2013-02-04

**Authors:** GUO-SHENG WANG, LIU YANG, HAI-FEN MA, YUAN GAO

**Affiliations:** 1 Department of Oncology, Beilun District People’s Hospital, The First Affiliated Hospital, College of Medicine, Beilun Branch of Zhejiang University, Ningbo, Zhejiang 315800;; 2 Department of Oncology, College of Medicine, The First Affiliated Hospital, Zhejiang University, Hangzhou, Zhejiang 310009;; 3 Department of Pathology, Beilun District People’s Hospital, The First Affiliated Hospital, College of Medicine, Beilun Branch of Zhejiang University, Ningbo, Zhejiang 315806;; 4 Department of Surgery, Division of General Surgery, Beilun District People’s Hospital, The First Affiliated Hospital, College of Medicine, Beilun Branch of Zhejiang University, Ningbo, Zhejiang 315800, P.R. China

**Keywords:** appendiceal cystadenoma, pseudomyxoma peritonei, public health medical resources

## Abstract

Appendiceal cystadenoma, the most common type of what has been generally termed ‘mucocele’ of the appendix, has unique clinical characteristics. In this study, three similar clinical manifestations of appendiceal cystadenoma are presented, with different subsequent management and diverse prognostic outcomes relating to the characteristics of the disease, the wishes of the patients, the medical workers and social factors. In this study, we provide details of three cases of appendiceal cystadenomas.

## Introduction

Appendiceal cystadenoma has unique clinical characteristics. It is a rare disease, accounting for 0.6% of appendectomy specimens (1), with an appendiceal carcinoid detection rate of 0.3–0.5%. A quarter of patients have no significant clinical manifestations (2). Appendiceal mucocele (AM) and appendiceal cystadenoma are distinct entities; the former is not a specific diagnosis, but a descriptive term for the dilation of the lumen of the vermiform appendix by an abnormal accumulation of mucous. These histopathologic lesions and mucoceles are defined as three types. One type is appendiceal cystadenoma. This is difficult to clinically identify and a surgical pathological diagnosis is required. The nosogenesis, pathology, biological activity, disease evolution, treatment points and prognosis (3) are different for each case. Due to the pressure retention and atypical cells, an appendix cystadenoma is often deformed and flat, resulting in a missed diagnosis. Pathologists are more likely to lean toward a diagnosis of appendiceal cystadenoma as opposed to appendiceal cyst. The appendiceal mucosa originates from the colon epithelium, so the lower gastrointestinal tract is vulnerable to the impact of the same tumor. It is reported that 20% of appendiceal cystadenomas are accompanied by a synchronous or metachronous colon tumor (5). Appendiceal cystadenoma is the required accurate preoperative diagnosis needed to prevent intraoperative rupture and predict malignant transformation (1,6) Appendix cystadenoma patients all have a different length of hospitalization, quality of life and prognosis due to the different timings of medical intervention and whether the cystadenoma is removed in its entirety. It’s prognosis as appendicitis requires a timely and complete resection, otherwise it will relapse into cystadenocarcinoma (4). Another possibility is a spontaneous rupture or an intraoperative rupture resulting in secondary pseudomyxoma peritonei (PMP), which has a clinical surgical detection rate of only 2/10,000 and a poorer prognosis (2). As a result, the prognosis of appendiceal cystadenoma relies upon the speed of treatment. This report of three cases of appendiceal cystadenoma seriously considers the timing of clinical intervention and the prognosis, as experienced in the primary hospital. This study was approved by the ethics committee of Beilun District People’s Hospital, the First Affiliated Hospital, Beilun Branch of Zhejiang University, Ningbo, China.

## Case report

### Case 1

**Figure 1:**
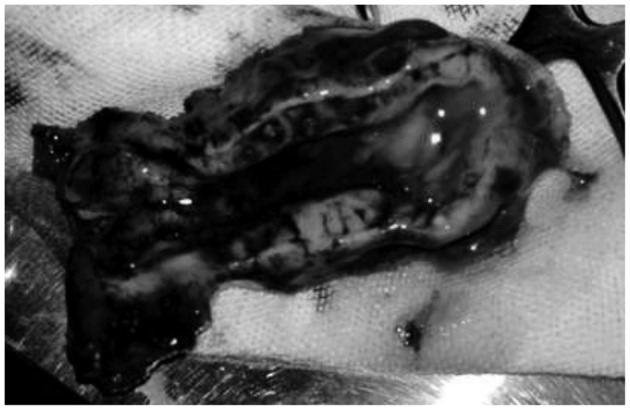
Yellowish jelly-like mucinous material was found after cutting the specimens.

A 79-year-old man with no notable medical or surgical history presented with a four-day-long history of distending pain in the right side of the abdomen. There was no palpable mass and no guarding or rebound tenderness was observed. Ultrasound showed a pathologic mass, 4 cm in diameter in the right side of the abdomen and the echo of the normal appendix was not displayed. Biochemistry laboratory results were within normal range. At surgery, a smooth mass was noted at the base of the appendix that continued into the cecum at the ileocecal junction. The tumor was not observed to be involved with the appendix, which, up to the base, was hard and encapsulated with no adhesions to the surrounding tissue. The frozen section of the mass was consistent with appendiceal cystadenoma. An appendectomy was performed. When the specimens were cut yellowish jelly-like mucinous material was observed (Fig. 1). The patient was discharged 7 days after surgery without discomfort and was followed up at 4 months. The patient has had no recurrence so far.

### Case 2

**Figure 2:**
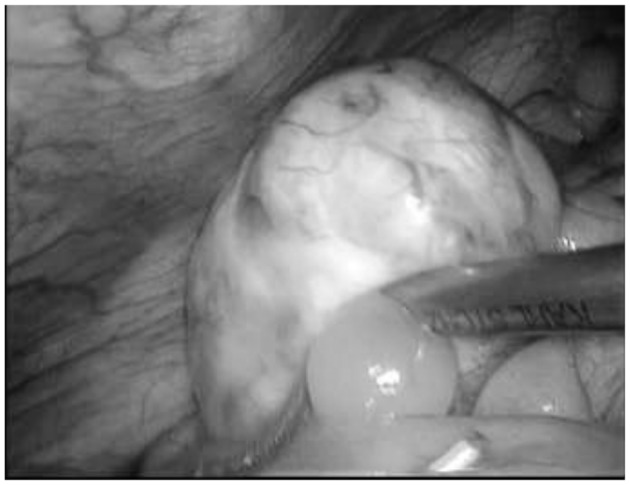
The capsule wall ruptured with an outflow of yellowish jelly-like mucus intraoperatively.

A 70-year-old woman was in good health until a large abdominal mass was detected in her right lower quadrant upon routine physical examination. Physical and laboratory examinations were within the normal range, but the patient had a raised carcinoembryonic antigen (CEA) level of 6.64 ng/ml. Ultrasound found a pathologic mass, which was consistent with appendiceal mucocele. The patient underwent a diagnostic laparoscopy. Intraoperatively, dense adhesions were not encountered in the right lower quadrant, but the capsule wall ruptured with an outflow of yellowish jelly-like mucus (Fig. 2). The patient received an irrigation of the peritoneal cavity instead of chemotherapy or hyperthermic chemotherapy. Ascites were observed in the abdominal drainage following surgery. The volume of drainage was assessed. There was 10 ml of yellowish mucus on day 5 after surgery, which increased to 15 ml on day 7. It was clean on day 18 after surgery and the patient was discharged. Sonography revealed seroperitoneum with maximum depth of ∼31 mm liquid anechoic area during the patient’s hospitalization. Few mucin-producing epithelial cells were found in the ascites on cytology. Biochemistry laboratory results of ascites revealed that Rivalta’s test was positive and a clinical diagnosis of PMP ascites type was made (5). At the last clinic visit, the patient reported with the chief complaint of abdominal distension. The patient’s abdomen was again enlarged and seroperitoneum was obtained again on sonographic studies. The patients refused to receive an ultraphonic guided puncture and was discharged after certain symptomatic approaches were performed.

### Case 3

**Figure 3:**
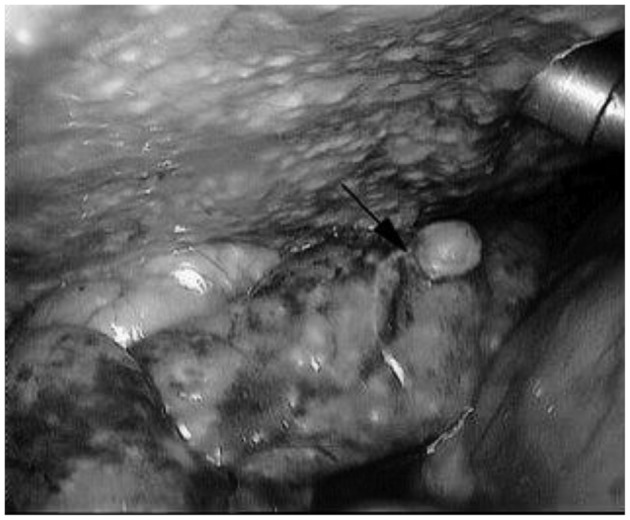
Diffusely light yellow nodules located at the surface of peritoneum without ascites and a piece of epiploic appendices with nodules located was cut (arrow).

**Figure 4:**
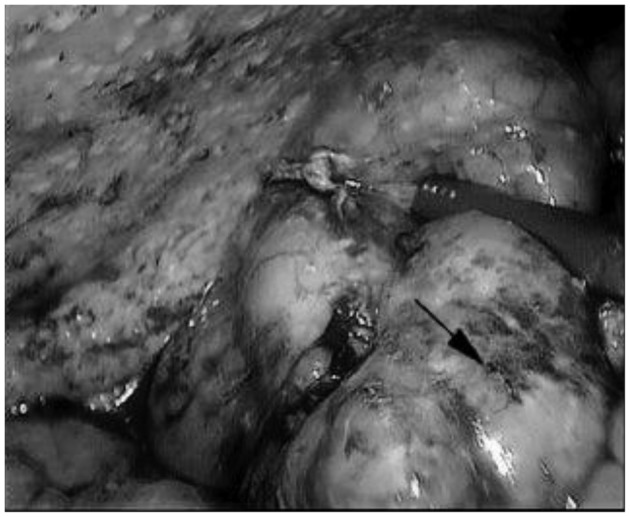
A firm irregular suspicious-looking tumor measuring approximately 2×2 cm was identified at the end of appendix (arrow).

**Figure 5:**
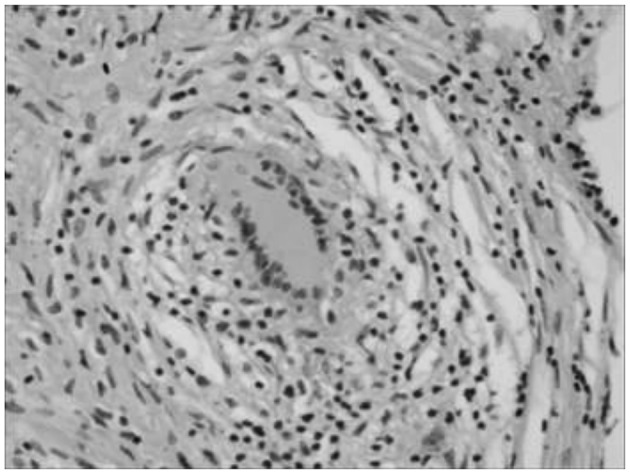
Intraoperative histopathologic examination demonstrated non-necrotizing granuloma (Hematoxylin & eosin; magnification, ×200).

**Figure 6:**
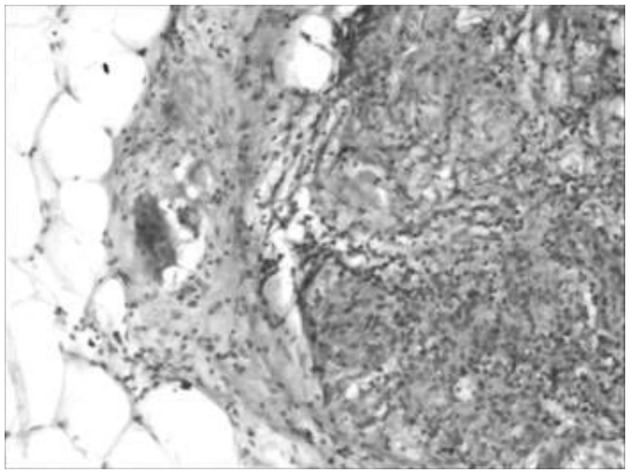
Acid-fast bacilli test was negative (Hematoxylin & eosin staining; magnification, ×200).

A 73-year-old female presented with a 1-month history of a distended abdomen, without nausea and vomiting. A computed tomography (CT) scan was obtained and revealed abnormal appendix enhancement, which was partially thickened at the surface of the liver and peritoneum. At this point, surgical options were discussed and the patient underwent diagnostic laparoscopy. Operative findings included diffusely light yellow nodules located at the surface of the liver and peritoneum without ascites (Fig. 3). There was a firm and irregular suspicious looking tumor measuring ∼2×2 cm at the end of appendix (Fig. 4), suspected to be an appendiceal cystadenoma after man-made rupture. A section of the epiploic appendices with nodules located was cut and intraoperative histopathologic examination was performed. This demonstrated non-necrotizing granuloma which was considered to be the same as the diffusely light yellow nodules located at the surface of the liver and peritoneum. There was little mucous membrane epithelium in the nodule on histopathologic examination, thus immunohistochemical methods were impossible. (Figs. 5 and 6). The medical team considered that a diagnosis of intestinal tuberculosis could not be eliminated, requiring medical therapy rather than surgery. In accordance with the wishes of the patient’s family, an appendectomy was not performed, even though a test for acid-fast bacilli was negative. A PPD skin test was inconclusive. After further investigation of the patient’s history, an ultrasound demonstrated a dilated and fluid filled appendix. The clinical diagnosis was ascites and low-grade appendiceal mucinous neoplasm. After 3 days of symptomatic approaches the patient improved and symptoms disappeared. The patient and her family refused to accept further checks and left the hospital. The patient remained healthy for 7 months with no treatment, but then had signs of bloating again. According to the history and clinical presentation, this case matched the diagnosis of PMP. The symptoms persisted but the patient’s family did not consent to further surgery due to her age and general condition. She was discharged for follow-up.

## Discussion

In China, appendiceal cystadenoma has its own characteristics (1). Located in an unremarkable organ, appendiceal cystadenoma is not considered to be serious. In large developing countries, such as China, community physicians often treat it as appendicitis. Diagnosis will occur in the first instance in community hospitals (2). Although the Chinese scientific community are willing to research tumor oncology to improve the survival time of months, they are yet to establish a set a countermeasures for appendiceal cystadenoma, even though this would be cost-effective and efficient (3). Chinese individuals often suffer from right lower abdominal pain. Many grass-roots hospitals treat this symptom collectively as ‘appendicitis’. If the pain is not severe it may be treated with antibiotics, which are easily obtained. The majority of sufferers of appendiceal cystadenoma are elderly individuals, who are more willing to take antibiotics rather than visit the Emergency Department (ED). These patients tend to only visit the ED if they have experienced multiple bouts of pain or if the pain becomes unbearable. This therapeutic strategy risks spontaneous rupture or malignant transformation of the appendiceal cystadenoma (4). In addition, the majority of patients wish to continue to work and do not have the time required to complete the follow-up treatment.

This study presents one case of a successful and complete resection, one case of intraoperative rupture due to the appearance of postoperative secondary PMP ascites, and one case of PMP nodal type. Three types of PMP, namely PMP ascites type, nodular type and mixed type (5–11) have been reported in the literature. It appears that these three types are part of a continuous process; the nodular type occurs late in the process and evolves from PMP ascites. The second of our cases did not receive cytoreductive surgery of hyperthermic chemotherapy which consists of warmed saline solution containing 30 mg mitomycin, 150 mg etoposide and 300 mg cisplatin, which is introduced into the peritoneal cavity for 60 min to maintain the abdominal temperature at 42–43°C. The patient also refused intraperitoneal chemotherapy and experienced postoperative secondary ascites at 2 weeks. Case 2 will be followed up to assess whether their progress follows the path of case 3. The three patients had very different costs of investments of medical resources (Table I).

Ultrasound is both pervasive and commonplace in China. When finding the right lower quadrant filled with fluid, ultrasound Doctors often consider appendix abscess rather than appendiceal cystadenoma. At this time a personal medical history should be taken to determine whether the patient has experienced pain in the past and how severe the pain has become. Even if the intraoperative pathological diagnosis is appendiceal cystadenoma, clinicians still prefer to believe that it is an appendiceal cyst, as this is more common. This results in: i) PMP: an appendix with an abscess requires a few months of conservative treatment before surgery. If the puncture fluid is mucus, the rupture caused by intervention may lead to PMP. Delayed surgery causes it to develop into secondary PMP. Case 3 presents a similar situation, since it is difficult to eliminate the minimal residual disease by chemotherapy and surgery. If the patient has a poorer prognosis, this disease produces canceration, which is more common in younger individuals and requires surgery as early as possible. ii) Rupture of PMP: assumption that the cause is a small cyst may lead to rupture of the PMP during surgery. Case 2 presents a similar situation.

Health care reform in China has reached a critical point. This economy has a large population and any small problem will be multiplied by the sheer size of the population and become exacerbated. Misunderstandings and treatment errors can result in a different prognosis of the same disease, which may result in an unreasonable allocation of medical resources.

With the global economy in distress, more direct and convenient high economic cost measures should be taken. Healthcare should be promoted in rural communities, particularly to the elderly. The grassroots level hospital is the first step in treating appendiceal disease and primary care doctors are a main force. They must therefore deepen their knowledge and increase their awareness of such presentations as described here. The difficulty of surgery will not increase, in comparison to treating appendicitis, if the treatment is not delayed. It may significantly affect prevention, treatment and amelioration of prognosis; it is therefore essential to understand the clinical manifestations of appendiceal mucinous tumor and its comprehensive pathological features, and to master the timely processing and proper disposal of the tumor. Finally, early diagnosis has important clinical significance as it can be complicated by malignant transformation, volvulus, intestinal necrosis, obstruction, intussusception, secondary infection, bleeding, PMP and even thrombosis of the iliac vein (12–14). Doctors therefore need to increase their understanding of recurrent right lower quadrant pain or mass, recurrent episodes of chronic appendicitis, or a history of appendiceal abscess.

## Figures and Tables

**Table I:** Details of three cases of appendiceal cystadenoma.

Case	Differential prognosis	Responsibility	Treatment observations	Spending on medical resources
1	Recovery	-	Timely Complete	Cost of surgery
2	Longer duration of hospitalization Follow-up care	Surgeon	Man-made rupture Non-effective remedial measures	Cost of surgery Longer duration of hospitalization Follow-up care
3	Longer duration and second hospitalization Follow-up care	Physician	Misdiagnosis the first time The delay led to spontaneous rupture and PMP	Twice the expenditure Follow-up care

PMP, pseudomyxoma peritonei.
